# Frozen Section Analysis in Submandibular Gland Tumors: Optimizing Intraoperative Decision-Making

**DOI:** 10.3390/cancers17050895

**Published:** 2025-03-05

**Authors:** Amir Bolooki, Felix Johnson, Anna Stenzl, Zhaojun Zhu, Benedikt Gabriel Hofauer

**Affiliations:** 1 Department of Otorhinolaryngology, Head and Neck Surgery, Klinikum Rechts der Isar, Technical University of Munich, 81675 Munchen, Germany; amir.bolooki@mri.tum.de (A.B.); zhaojun.zhu@mri.tum.de (Z.Z.); 2 Department of Otorhinolaryngology-Head and Neck Surgery, Medical University of Innsbruck, Anichstr. 35, 6020 Innsbruck, Austria

**Keywords:** salivary gland tumor, frozen section, submandibular gland, gland excision, pleomorphic adenoma

## Abstract

**Introduction**: With around 25 different salivary gland tumor entities described by the World Health Organization, the correct preoperative identification of masses as benign or malignant remains a challenge. If preoperative needle biopsy is inconclusive, frozen section analysis is a possible alternative for accurate histological identification. The purpose of our study was to evaluate the diagnostic effectiveness of frozen section performed for primary submandibular gland masses. **Methods**: In addition to acquiring epidemiological data from patients who underwent submandibular gland excision over a 20-year period, we analyzed the diagnostic effectiveness of frozen section performed for submandibular gland masses. We also examined the impact of frozen section on overall survival. Furthermore, we investigated whether there was an impact on the surgical revision rate for malignant submandibular gland masses that required additional neck dissection within the submandibular triangle. **Results**: Frozen section was performed for 54 submandibular gland tumors, with a specificity of 100% and a sensitivity of 81.3%. Frozen section was conducted in 12 cases of primary salivary gland malignancies, of which 9 cases were identified correctly. In three cases, the frozen section results were inconclusive. We calculated a relative risk reduction of 27% for revision surgery by performing frozen section. There was no significant association between frozen section results and overall survival. **Conclusions**: Frozen section demonstrates a significant reduction in the need for revision surgery. With a specificity of 100%, frozen section is especially suited to identifying benign masses. It is a valid diagnostic tool when preoperative sampling is not possible or is inconclusive.

## Simple Summary

Because of the large variety of salivary gland tumor entities, correct pathological classification is essential to planning further diagnostic and therapeutic steps. Since needle biopsy results are not always conclusive, we tried to evaluate the effectiveness of frozen section (immediate intraoperative pathological evaluation) for such salivary gland masses through a retrospective data collection over a 20-year period in our hospital. In our study, frozen section showed a specificity of 100% and a sensitivity of 81.3%. In addition, we calculated a relative risk reduction of 27% for revision surgery for malignant salivary gland masses, highlighting the benefit of frozen section, especially in cases where preoperative biopsy was not possible or inconclusive.

## 1. Introduction

With approximately 25 different salivary gland tumor entities described by the World Health Organization, the preoperative identification of masses through physical examination and imaging alone remains a challenge [1,2]. In addition to solid masses, a variety of infectious or systemic diseases can manifest in the salivary glands [3]. Nevertheless, the preoperative evaluation of malignancy risk is key to developing an adequate surgical plan. If malignancy is detected only after gland excision, an additional neck dissection may need to be performed during a second surgery, depending on the tumor entity and TNM staging. Considering the higher malignancy rates within the submandibular glands (approximately 43%) compared to parotid lesions, the correct and timely histological identification of submandibular gland lesions is especially important for improving overall outcomes [4,5,6,7].

Apart from the mental toll on a patient with a new tumor diagnosis, surgical revision can also be technically challenging for the physician. The anatomy of the head and neck is complex due to the various nerve structures, not to mention the elevated risk during revision surgery caused by altered anatomy and scarring from the initial gland excision. One of the main structures at risk at level Ib during initial gland excision, and especially during surgical revision, is the marginal mandibular branch of the facial nerve [8,9,10,11]. Injury to this structure can lead to a considerable reduction in the quality of life for patients, resulting in aesthetic deformities (such as an asymmetric face) or functional impairments (such as salivary incontinence) [12,13]. This highlights the importance of identifying salivary gland masses as benign or malignant before or during gland excision to avoid revision surgery.

While the ESMO guidelines recommend fine needle aspiration cytology as the initial form of sampling, many physicians prefer core needle biopsy due to its higher diagnostic yield. A large meta-analysis involving 5647 patients demonstrated a specificity ranging from 78.6% to 100% and a sensitivity of between 72.4% and 88% for fine needle aspiration [14]. Core needle biopsy is typically performed in cases where malignancy is suspected and has an estimated sensitivity of about 92–94% and a specificity of between 99% and 100% [15,16,17,18,19]. However, core needle biopsy of some malignant tumors has been shown to be inferior; for example, the sensitivity of core needle biopsy for lymphomas was 66%, with a specificity of 100%, in a prospective study [20]. Furthermore, in some cases, it is not possible to obtain enough histological material to make a definitive preoperative diagnosis. Considering the advantages and disadvantages of fine needle aspiration cytology and core needle biopsy described in the literature, frozen section could be a possible alternative for acquiring samples for histological workup and for making intraoperative decisions to escalate the surgical approach [21,22,23,24]. The decision to perform a frozen section analysis depends on features that typically present in cases of malignant neoplasms.

The main goal of this study was to investigate the diagnostic effectiveness of frozen section performed for submandibular gland lesions and its impact on the surgical revision rate in cases of malignancy.

## 2. Material and Methods

The German procedure classification for submandibular gland excision was used to analyze cases of solid submandibular gland masses, both benign and malignant, at our hospital over a 20-year period (2002–2021). We collected the epidemiological data using the electronic patient chart. Cases were divided according to tumor type. We placed special focus on the cases in which a frozen section was performed. These cases were divided into three groups depending on their results: malignant, not malignant, and unclear. Specificity and sensitivity were calculated. The frozen section results were compared to the gold standard, which is the definitive pathological report. Furthermore, we investigated whether the result of the frozen section could be linked to the surgical revision rates using the chi-square method, including Yates’s correction for continuity due to our low case numbers. The relative risk reduction was calculated. A *p*-value of <0.05 was considered statistically significant. We compared the overall survival of the most common primary salivary gland malignancies, particularly between patients with and without frozen section, using a Kaplan–Meier analysis. The study protocol was in accordance with the Declaration of Helsinki. The Institutional Review Board of the Medical Faculty of the Technical University of Munich reviewed and approved the protocol (2023-108-S-SR). Statistical analysis was performed using version 28.0 of the Social Sciences Statistical Package software (SPSS, Chicago, IL, USA). Descriptive data are reported as mean ± standard deviation, unless otherwise stated. The normal distribution of the variables was tested using the Shapiro–Wilk test. A *p*-value of <0.05 was considered statistically significant.

## 3. Results

**Figure 1:**
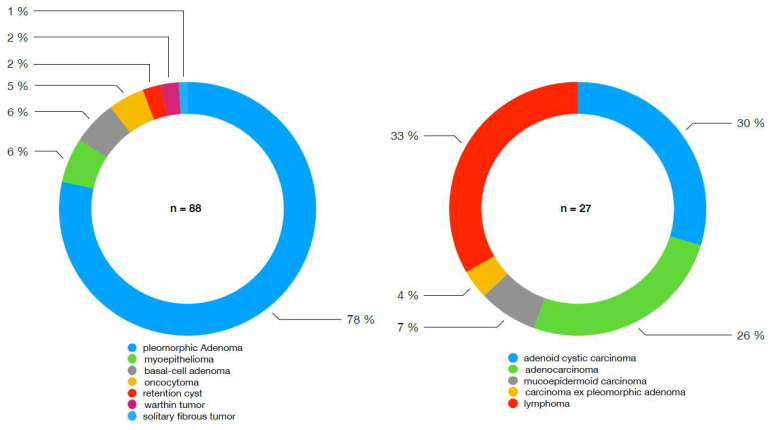
Comparison of benign and malignant entities. No statistical test performed.

A total of 115 gland excisions were performed for solid submandibular gland masses. Benign entities accounted for 76.5% (*n* = 88) of the cases, while malignant tumors comprised 23.5% (*n* = 27). The composition of these entities is illustrated in Figure 1.

**Figure 2:**
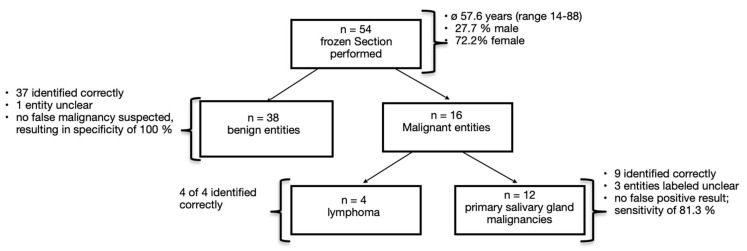
Frozen section entities.

A frozen section was performed for 54 submandibular gland tumors. This cohort consisted of 28% male and 72% female patients, with a mean patient age of 57.6 years (±15.2). A frozen section was conducted for 38 of the 88 benign lesions, and 37 of these were identified correctly. Only one entity (pleomorphic adenoma) was labeled as “unclear” by the frozen section (see Figure 2). For the 27 malignant tumor cases, a frozen section was performed in 16 instances. These included 4 cases of lymphoma and 12 cases of primary salivary gland malignancies. The frozen section was accurate in all four cases of lymphoma. In 9 of the 12 primary salivary gland malignancy cases, malignancy was correctly identified. One of these nine cases involved a patient who underwent gland excision solely to obtain a histological sample, while already suspecting distant metastasis based on preoperative imaging. In the other eight cases, an additional neck dissection was performed during the same surgery. In three cases of primary salivary gland malignancies (adenoid cystic carcinoma, adenocarcinoma, and carcinoma ex pleomorphic adenoma), the frozen section results were unclear. Consequently, an additional neck dissection had to be performed in a second surgery after the final pathological report confirmed malignancy. Due to the absence of false positive results, no unnecessary neck dissection was performed. In our study, the frozen section demonstrated a specificity of 100% and a sensitivity of 81.3%. In 29 of the 38 (76.3%) correctly identified benign tumor cases, the frozen section even predicted the exact entity. In contrast, among the nine primary salivary gland malignancies correctly identified by the frozen section, five were also classified by the correct histological subtype (1 adenocarcinoma and 4 adenoid cystic carcinomas).

**Figure 3:**
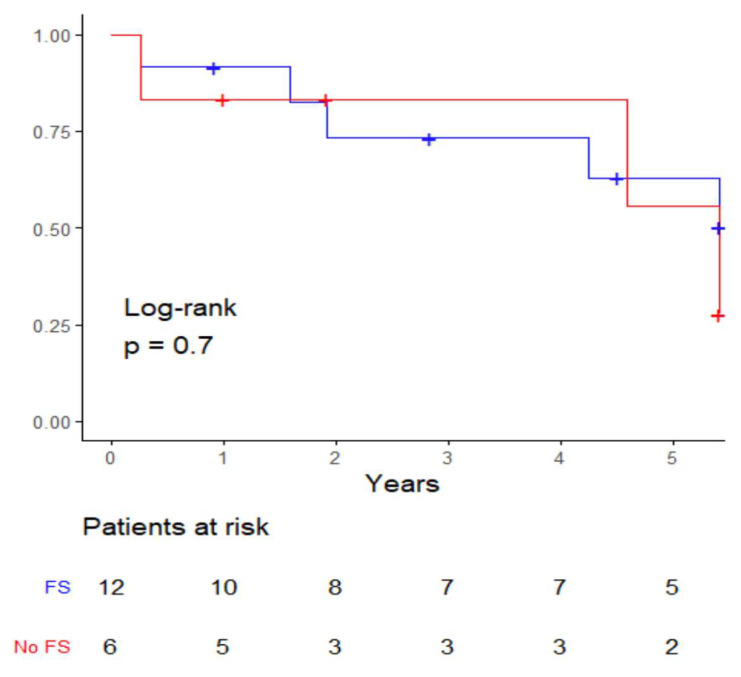
Kaplan–Meier analysis of frozen section (FS) vs. non-frozen section (no FS).

Core needle biopsy was not performed for any of the cases where a frozen section was conducted. There was no significant correlation between patients with primary salivary gland malignancies who underwent frozen section and those who did not, in terms of overall survival (see Figure 3). Additionally, there was no significant correlation when comparing the overall survival of the two most common primary salivary gland malignancies in our study (adenoid cystic carcinoma and adenocarcinoma).

A statistically significant association (*p*-value < 0.05) between “surgical revision needed and non-frozen section“ and “association between frozen section and not-needed surgical revision” was demonstrated not only by the chi-square method (*p*-value of 0.0041) but also by Yates’s correction for continuity (*p*-value of 0.0181). We calculated a relative risk reduction of 27% for revision surgery by performing a frozen section beforehand (see Table 1).

## 4. Discussion

The main goal of our study was to investigate the impact of frozen section on the surgical revision rate. In our cohort, no benign mass was falsely identified as a malignant tumor during frozen section, resulting in a specificity of 100%. For submandibular gland malignancies, frozen section had a sensitivity of 81.3%. Both numbers resemble the results of other studies investigating the effect of frozen section [8,10,11]. While calculating the relative risk reduction, only 17 of 18 primary submandibular gland malignancies were included. The excluded case was a patient with M1 status, who did not require surgical revision. The specimen of the M1 patient also underwent frozen section. Seventy-three percent (*n* = 8) of cases in which frozen section was performed for primary submandibular gland malignancies and additional neck dissection was needed were operated on during one surgery. All patients with primary submandibular gland malignancies, where frozen section was not performed (*n* = 6), required revision surgery. Revision surgery showed a significant relative risk reduction of 27% when frozen section was performed. With a specificity of 100% and no false positive cases, there appear to be no disadvantages in terms of unnecessary radical operations for patients. However, looking at our cohort, which included the aforementioned M1 patient, it is important to rule out distant metastasis through staging imaging beforehand if malignancy is suspected. Only then can the physician expand the surgery if frozen section suggests malignancy. In the case of M1 status, palliative radiotherapy or systemic treatment needs to be discussed [23]. The specificity of 100% for frozen section is especially useful in cases of submandibular masses, as they usually show higher malignancy rates [25].

Frozen section can also be used not only to identify the entity itself but also to evaluate the margins of resection, perineural invasion, and possible lymph node metastasis. Concepts of elective neck dissection vary across hospitals. The new salivary gland malignancy guideline from The European Society for Medical Oncology recommends a therapeutic neck dissection for salivary gland carcinomas with clinically or radiologically evident lymph node metastases (cN+), as well as for high-risk tumors, including high-grade histologies such as salivary duct carcinoma, high-grade mucoepidermoid carcinoma, adenocarcinoma, and high-grade acinic cell carcinoma. Adenoid cystic carcinoma with extensive perineural invasion or locally advanced (T3/T4) tumors may also warrant elective neck dissection due to the increased risk of occult metastases [23]. Postoperative radiotherapy is indicated for submandibular gland carcinomas in the following cases: pT3/T4 tumors, intermediate-grade or high-grade tumors, adenoid cystic carcinoma (ACC), positive resection margins (R+), lymph node metastases (N+), bone invasion, lymphovascular invasion (L1), venous invasion (V1), perineural invasion (Pn1), and recurrent tumors. These factors increase the risk of local recurrence, making radiotherapy essential to improve local control and overall prognosis. The role of chemotherapy is limited and is mainly considered in palliative situations or within clinical trials [23].

**Figure 4:**
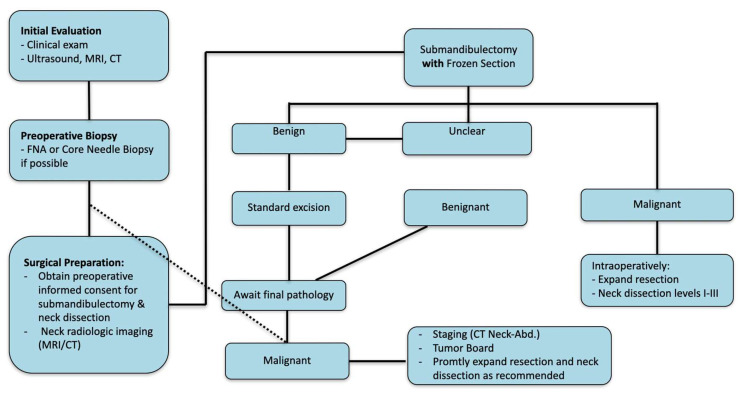
Diagnostic pathway to be followed in cases of submandibular gland masses.

If preoperative sampling of the tumor is unsuccessful, revision surgery after gland excision is almost guaranteed, given all these complex and widely varying surgical concepts, even if malignancy is suspected preoperatively. Frozen section could help identify malignant lesions, allowing for the expansion of resection margins. Additionally, level Ib could always be included in the gland resection. If pathologists suspect high-grade carcinomas or malignancy in specifically sampled lymph nodes, the physician could further expand the neck dissection (levels I–III or even up to level V for N+ patients), positively impacting the rate of revision surgery [23,26,27,28,29]. Figure 4 shows a possible pathway to incorporating frozen sections into the diagnostic process of submandibular gland masses recommended by the ESMO. A possible limitation for frozen section biopsy would be that in some cases malignancy is suspected but not further specified into a specific subtype (high grade vs. low grade). If the suspected malignancy cannot be further classified, the intraoperative therapeutic consequences of the frozen section results are limited since the extent of the neck dissection needed in case of malignancy varies depending on the tumor grading [30,31].

Even though overall survival does not seem to be influenced by frozen section, in our opinion, patients could benefit from the lower number of surgeries performed and reduced time spent in the hospital in terms of quality of life [32,33,34,35]. A possible tool to evaluate such an impact would be a patient-reported outcome measure (e.g., questionnaires) focusing on the postoperative pain, functional recovery, and psychological burden of the prolonged hospital stay in the case of revision surgery [36,37].

Furthermore, if revision surgery is averted, the physician benefits from the intact head and neck anatomy, which remains untouched by previous surgery, allowing for better identification and preservation of key anatomical structures such as facial nerve branches [10,38,39]. However, clear conclusions cannot be drawn from the Kaplan–Meier analysis alone due to the very small case numbers in each cohort (*n* < 10) and because the majority of patients in our study were still eligible for definitive surgery and thus for a curative therapeutic concept most of the time, as cases were identified using the German procedure classification for submandibular gland excision. Additionally, in the cases of revision surgery, no relevant postoperative impairment of facial movement was observed, even though a few surgical reports pointed out scar tissue from the initial operation, challenging the argument of an elevated risk for facial nerve damage.

Ultrasound examination is especially suited to assessing the cervical lymph node status as well as the main submandibular lesion [40]. Preoperative diagnostics for salivary gland tumors often include a biopsy in addition to imaging. Regarding histological sampling, multiple studies have been published comparing fine needle aspiration cytology and core needle biopsy. In general, core needle biopsy seems to be superior to fine needle aspiration cytology regarding diagnostic yield. However, there is still room for error. Some tumor entities, such as carcinoma ex pleomorphic adenoma, pose special challenges for the pathologist. It is possible for samples taken by core needle biopsy to not include the malignant focus needed for diagnosis due to the limited amount of tissue acquired. In contrast, frozen section biopsy is performed on the whole removed specimen, providing the pathologist with significantly more tissue to work with [10,21,22,23,41,42]. It should be noted that, if possible, preoperative sampling of the tumor is preferred since it allows for more adequate planning of further diagnostic and surgical steps. However, depending on the tumor localization and the diagnostic yield of the procedure used for sampling, a sufficient preoperative diagnosis of the entity is not always possible. Especially if fine needle biopsy is performed, the pathological report often suspects malignancy while not definitely proving it, in which case frozen section would provide additional information. Hence, frozen section is especially suited as a confirmatory tool [30,31].

In contrast to parotid carcinomas, the rate of functional deficits of the facial nerve (which highly indicates malignancy) is lower for submandibular gland malignancies. Therefore, preoperative evaluation of the malignancy rate in submandibular gland tumors through physical examination alone can be tricky, as the most common symptom at first presentation is simply a growing mass. This highlights the key role of frozen section, which clarifies the histological entity intraoperatively when malignancy may not have been suspected initially [23,43,44,45].

Cases of lymphoma were only included if the pathological report described infiltration of the gland itself, excluding solely extraglandular manifestations. Even though extranodal manifestation of lymphomas inside salivary glands is rather rare, with the parotid gland being the most commonly affected gland, manifestation inside the submandibular gland is also described in the literature [46,47]. Histological sampling is needed to identify the correct lymphoma subtype. While fine needle aspiration seems like a less invasive option, it only shows low sensitivity rates and mainly provides information on the cytology of the specimen. Core needle biopsy shows better results, but there are still obstacles to overcome. There is a limited diagnostic yield for cases of composite lymphomas or if the specimen shows nodal necrosis. Furthermore, if smaller biopsy needles are used, the diagnosis of Hodgkin’s lymphoma becomes more challenging due to the paucity of Reed–Sternberg cells. Excisional biopsy is still regarded as the gold standard, as it enables immunohistochemical staining and full assessment of the nodal structure, which is relevant for correct subtyping. In our study, frozen section showed a sensitivity of 100% (*n* = 4/4) for the diagnosis of lymphomas. Since open biopsy is recommended, frozen section can be used to quickly identify hemato-oncological neoplasms. While detailed staining and subtyping take time, the intraoperative discovery of a hemato-oncological neoplasm can facilitate early referral of the patient to the oncology department, streamlining further steps [46,48,49,50]. Additionally, intraoperative diagnosis of a hemato-oncological neoplasm could avert extensive surgery in general. This aspect also applies to inflammatory diseases such as tuberculosis [51,52].

Due to the retrospective nature of our study, it is not possible to retrace how physicians determined which cases were suitable for frozen section. Since needle biopsy was not performed in any of the frozen section cases, no clear comparison between both diagnostic tests could be drawn in our study. An approach to further evaluate the usefulness of frozen section in the clinical setting would be to correlate core needle biopsy results with the definitive pathological report and compare these results to the frozen section cohort and their accordance with the definitive pathological report. The main goal would be to identify the superior diagnostic approach. Furthermore, cases in which core needle biopsy is negative or inconclusive should be correlated with the frozen section results to investigate whether frozen section could complement core needle biopsy, thereby further benefiting the current literature.

## 5. Conclusions

In cases where the diagnosis of a submandibular tumor cannot be determined preoperatively, we recommend performing an intraoperative frozen section due to the high risk of malignancy associated with submandibular gland tumors. Additionally, with a specificity of over 90% in our cohort and the published literature, frozen section analysis is particularly well suited for identifying benign masses in the submandibular gland. The main limitation of our study is the relatively small number of primary salivary gland malignancies.

## Figures and Tables

**Table 1:** Surgical revision comparison.

	Revision Yes	Revision No	
Frozen section	3 (27% of patients with FS performed)	8 (73% of patients with FS performed)	11
Non-frozen section	6	0	6 (100% of patients without FS performed)
	9	8	17

## Data Availability

The data that support the findings of this study are available from the corresponding author upon reasonable request.
